# Temporal evolution of neurovascular coupling recovery following moderate‐ and high‐intensity exercise

**DOI:** 10.14814/phy2.14695

**Published:** 2021-01-19

**Authors:** Joel S. Burma, Alannah Macaulay, Paige V. Copeland, Omeet Khatra, Kevin J. Bouliane, Jonathan D. Smirl

**Affiliations:** ^1^ Concussion Research Laboratory Faculty of Health and Exercise Science University of British Columbia Kelowna BC Canada; ^2^ Sport Injury Prevention Research Centre Faculty of Kinesiology University of Calgary Calgary AB Canada; ^3^ Human Performance Laboratory Faculty of Kinesiology University of Calgary Calgary AB Canada; ^4^ Hotchkiss Brain Institute University of Calgary Calgary AB Canada; ^5^ Alberta Children’s Hospital Research Institute University of Calgary Calgary AB Canada; ^6^ Libin Cardiovascular Institute of Alberta University of Calgary AB Canada; ^7^ School of Health Sciences, Nuclear Medicine British Columbia Institute of Technology Burnaby BC Canada; ^8^ Faculty of Medicine University of British Columbia Vancouver BC Canada

**Keywords:** acute recovery, cerebral blood flow, high‐intensity interval training, moderate‐intensity continuous training, neurovascular coupling, posterior cerebral artery

## Abstract

**Purpose:**

Studies examining neurovascular coupling (NVC) require participants to refrain from exercise for 12–24 hours. However, there is a paucity of empirical evidence for this restriction. The objectives for this study were to delineate the time‐course recovery of NVC metrics following exercise and establish the NVC within‐ and between‐day reliability.

**Methods:**

Nine participants completed a complex visual search paradigm to assess NVC via transcranial Doppler ultrasound of the posterior cerebral artery blood velocity (PCA). Measurements were performed prior to and throughout the 8‐hour recovery period following three randomized conditions: 45 minutes of moderate‐intensity exercise (at 50% heart‐rate reserve), 30 minutes high‐intensity intervals (10, 1‐minute intervals at 85% heart‐rate reserve), and control (30 minutes quiet rest). In each condition, baseline measures were collected at 8:00am with serial follow‐ups at hours zero, one, two, four, six, and eight.

**Results:**

Area‐under‐the‐curve and time‐to‐peak PCA velocity during the visual search were attenuated at hour zero following high‐intensity intervals (all *p* < 0.05); however, these NVC metrics recovered at hour one (all *p* > 0.13). Conversely, baseline PCA velocity, peak PCA velocity, and the relative percent increase were not different following high‐intensity intervals compared to baseline (all *p* > 0.26). No NVC metrics differed from baseline following both moderate exercise and control conditions (all *p* > 0.24). The majority of the NVC parameters demonstrated high levels of reliability (intraclass correlation coefficient: >0.90).

**Conclusion:**

Future NVC assessments can take place a minimum of one hour following exercise. Moreover, all metrics did not change across the control condition, therefore future studies using this methodology can reliably quantify NVC between 8:00am and 7:00 pm.

## Graphical abstract

Previous studies examining neurovascular coupling (NVC) metrics, required participants to refrain from exercise for 4‐24 hours prior to data collection, despite a lack of objective evidence for this time restriction. The results in the current investigation demonstrated that the NVC response was attenuated immediately following high‐intensity exercise within the posterior cerebral artery (PCA); however, the NVC response was comparable to baseline values one‐hour following exercise. Additionally, NVC metrics displayed high levels of within‐ and between‐day reliability across the control condition (8:00am – 7:00pm) when using a complex visual scene search paradigm (“Where’s Waldo”).

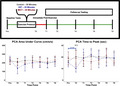

## 1 INTRODUCTION

Maintaining the supply of oxygen and nutrient delivery to the brain is of utmost importance while at rest and during exercise, as the brain has a limited capacity for substrate storage (Willie, Cowan, et al., 2011). To accommodate the metabolic demands associated with cerebral cortex neural activity, a coordinated response occurs providing increased cerebral blood flow to the working cortical regions in a process known as neurovascular coupling (NVC) (Smirl et al., 2016; Wright et al., 2017). This response can be quantified through transcranial Doppler ultrasound (TCD) to determine cerebral blood velocity (CBV) in the posterior cerebral artery (PCA), which is the main cerebral artery bed responsible for supplying the brain's primary visual processing centers (Willie, Colino, et al., 2011). Therefore, during visually stimulating protocols, such as a visual search paradigm, CBV will increase to match the metabolic demands associated with the visual cortex activation levels (Smirl et al., 2016).

The impact of a bout of exercise on the NVC response remains controversial. For example, one study measuring the NVC response *during* cycling exercise at 70% of maximal heart rate reported no difference in NVC metrics when compared to resting values (Willie, Cowan, et al., 2011). This finding confirmed a previous investigation that also found no change in NVC with a variety of orthostatic conditions (Azevedo et al., 2007). Additionally, a study examining NVC *during* a 30% maximal voluntary contraction handgrip exercise, found no change in the NVC response, even with an increase in absolute baseline PCA blood velocity (Yamaguchi et al., 2014). Contrarily, other studies have found the NVC response is attenuated with moderate exercise above a heart rate of 140 beats per minute (Yamaguchi et al., 2015a) and during a progressive cycling protocol to exhaustion (Yamaguchi et al., 2015b). Furthermore, while several studies have investigated NVC alterations *during* exercise (Willie, Cowan, et al., 2011; Yamaguchi, Ikemura, & Hayashi, 2015; Yamaguchi, Ikemura, Kashima, et al., 2015; Yamaguchi et al., 2014), there is very little research examining these metrics both immediately (<15 minutes) and acutely (several hours) into *recovery*.

Currently, experiments investigating NVC metrics require subjects to abstain from any physical activity for four (Williams et al., 2017), six (Yamaguchi, Ikemura, & Hayashi, 2015; Yamaguchi, Ikemura, Kashima, et al., 2015; Yamaguchi et al., 2014), twelve (Caldwell et al., 2018; Smirl et al., 2016; Wright et al., 2017), or twenty‐four hours (Phillips et al., 2013, 2014; Willie, Cowan, et al., 2011) prior to participation, to safeguard CBV and aim to have cerebrovascular metrics return to their baseline levels. Ultimately, this mitigates the influence acute exercise may have on the data being collected. However, based on the knowledge of the authors, these specific time restrictions with respect to the cerebrovasculature following an exercise bout are not informed by any firm empirical evidence. Further, previous literature has demonstrated that clinical conditions, such as sport‐related concussion, alter NVC metrics in both the acute (Wright et al., 2017) and chronic phase of injury (Sharma et al., 2020). However, the acute measures were collected ~72 hours following injury (Wright et al., 2017). Additionally, it has been found that repetitive subconcussive head impacts alter the cerebrovasculature and the NVC response (Smirl et al., 2020). Thus, for future studies seeking to obtain NVC metrics using on‐site mobile testing laboratories; it is imperative to establish an objective timeframe, NVC assessments must wait to ensure the acute effects of exercise do not confound these measurements. As well, it is important to understand how NVC parameters are impacted by the time of day, as an individual can sustain a concussion at any time point across a day.

The one study that examined the prolonged effect exercise has on the NVC response, had participants engage in a single bout of exhaustion, which consisted of a ramp test to exhaustion (Yamaguchi, Ikemura, & Hayashi, 2015). Additionally, the NVC metrics were derived using a reversed checkboard stimulus that consisted of two eyes open/closed cycles over two minutes (Yamaguchi, Ikemura, & Hayashi, 2015). However, current NVC recommendations state that in order to maximize the signal to noise ratio, a minimum of five cycles should be performed (Phillips et al., 2015).

Therefore, the purpose of this study was to further the previously described research by: 1) examining how two different acute bouts of exercise (moderate‐intensity continuous training (MICT) and high‐intensity interval training (HIIT)) affect NVC metrics, rather than a single ramp protocol; 2) comprehensively examining the time‐course recovery of the NVC response using eight visual cycles, rather than two cycles; 3) comparing the exercise values to a resting control condition to understand if any changes are attributable to temporal changes rather than interventional, which also enables the computation of the between‐day reliability of NVC metrics; and 4) examining how diurnal variation impacts the NVC response across the control day between 8:00am and 7:00 pm. It is hypothesized that the NVC response will be attenuated immediately following both exercise protocols, based upon the known physiological alterations that occur with exercise (Burma et al., 2020a; Ide & Secher, 2000; Secher et al., 2008; Seiler et al., 2007; Smith & Ainslie, 2017; Smith et al., 2014). Ultimately, if the current four‐ to twenty‐four hour timeline can be reduced, it will increase the ability to test active individuals; as well as certain conditions that may occur within sporting environments (e.g., sport‐related concussion).

## 2 METHODS

A total of 20 healthy young participants (male = 12) (age: 24 ± 4 years, Body Mass Index: 26 ± 5 kg/cm^2^) were recruited from the university setting to partake in this study, where nine of these participants (male = 7) completed all three arms of the current investigation (age: 26 ± 5 years, Body Mass Index: 25 ± 4 kg/cm^2^). Within this investigation, healthy was operationally defined as nonsmoking individuals who had a normative resting heart rate (i.e., ~60–80 bpm) (McArdle et al., 2014), a normal blood pressure (~120/80) (Lin et al., 2016), active individuals who were classified as having a normal or slightly overweight BMI (due to elevated muscle mass), and regularly adhered to American guidelines for physical activity (Piercy et al., 2018). Potential participants were excluded based upon a history of any neurological, cerebrovascular, cardiorespiratory, or musculoskeletal complications. Finally, to counteract any effect hormonal variation plays within females due to the menstrual cycle, female participants came in for testing on days three to seven of the early follicular phase of their menstrual cycle, as hormones are relatively stable during this window (Boivin & Shechter, 2010).

**FIGURE 1:**
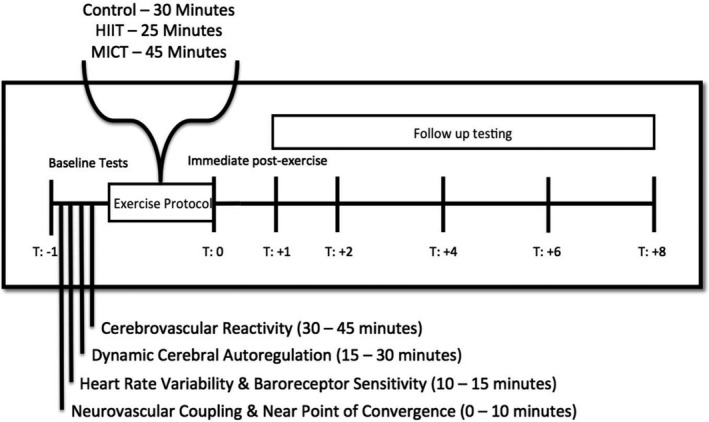
The order of testing of each cerebrovascular and cardiovascular assessment before and after a randomly selected exercise condition. To ensure the cerebrovascular reactivity assessment did not impact the next hour neurovascular coupling assessment, a 15‐minute washout period was implemented. Testing was initiated at 8:00am and was fully completed by 7:00 pm each testing day. Reprinted with permission (Burma et al., 2020a, 2020d; Burma, Copeland, Macaulay, Khatra, Wright, et al., 2020; Burma, Macaulay, Copeland, et al., 2020)

Using a randomized cross‐over cohort design, participants completed three conditions (control, HIIT, and MICT) (Figure 1). To avoid potential confounding from previous testing sessions, all interventions were separated by a minimum of three days. For each participant, all data were collected within two months of the first testing session. On all three days, NVC metrics were assessed at baseline (pre) before the randomized condition and repeatedly after the three conditions (i.e., MICT, HIIT, and control) at hours zero, one, two, four, six, and eight. To negate the potential influence of diurnal variation, testing was initiated at the same time each day (8:00am) (Conroy et al., 2005). All testing protocols were explained and demonstrated to ensure each participant was familiar with the procedures. Following the previous NVC literature guidelines, participants abstained from exercise, caffeine, and alcohol for at least 12 hours before the study (Caldwell et al., 2018; Smirl et al., 2016; Wright et al., 2017). To control for any dietary impacts between the testing conditions, participants consumed two Gatorades (Gatorade Perform, PepsiCo; 150 calories each) and two meal replacement drinks (Vanilla Nutrition Shake, Kirkland Signature; 210 calories each) between time points two and four. Furthermore, they had access to water and were able to use the washroom when required.

This study was a subsection of a larger study examining the postexercise effects MICT and HIIT have on visual (near point of convergence), cardiovascular (cardiac baroreceptor sensitivity and heart rate variability (Burma et al., 2020a)), and cerebrovascular function (neurovascular coupling, cerebrovascular reactivity (Burma, Macaulay, Copeland, et al., 2020) and dynamic cerebral autoregulation (Burma et al., 2020d; Burma, Copeland, Macaulay, Khatra, Wright, et al., 2020)) (Figure 1). This investigation examines the effect different modalities of exercise have on NVC components and the duration these effects persist into recovery and the within‐day variability in NVC measures. As shown in Figure 1, the various assessments were conducted in a strategic order to minimize the likelihood the preceding experimental protocol impacted the one following. Additionally, there was a 15‐minute time buffer between the end of the cerebrovascular reactivity protocol and the start of the NVC assessment, which was sufficient to ensure the reactivity did not impact the NVC. This study was approved by the University of British Columbia clinical ethics review board (H16‐00507) and took place from November 2016 to August 2018. Participants provided written informed consent prior to commencement within the study.

### 2.1 Instrumentation

A TCD was employed to index cerebral blood flow using 2‐MHz ultrasound probes (Spencer Technologies, Seattle, WA, USA) over the temporal acoustic windows, to determine left PCA and right MCA blood velocities. As this was a substudy of a larger investigation, unilateral measures were recorded to ensure the probe insonation angle and/or location did not change across any of the interventions. Once vessels were identified and confirmed using carotid artery compressions and visual tasks (Willie, Colino, et al., 2011), probes were locked into place using a fitted head‐frame (Spencer Technologies, Seattle, WA, USA). Heart rate was obtained through a three‐lead electrocardiogram. A finger photoplethysmography with a brachial cuff was used to measure beat‐to‐beat mean arterial pressure (MAP) and was corrected to the height of the heart (Finometer PRO, Finapres Medical Systems, Amsterdam, Netherlands). An inline gas analyzer (ML206, ADInstruments, Colorado Springs, CO) was utilized to monitor respiration rate and the end‐tidal partial pressure of carbon dioxide (P_ET_CO_2_) via a mouthpiece, which was calibrated with known gas concentrations prior to each data collection and corrected to the atmospheric pressure in the laboratory. Data were sampled at 1000 Hz (PowerLab 8/30 ML880, ADInstruments), time‐locked, and stored for offline analysis with commercially available software (LabChart version 7.1, ADInstruments).

### 2.2 Experimental Protocols

As previously described, NVC responses were quantified using a complex scene search paradigm (*Where's Waldo*) (Smirl et al., 2016). This was utilized as this paradigm has been shown to provoke a robust and reliable NVC response in the PCA (~30 percent increase) and the MCA (~8 percent increase (Smirl et al., 2016). In brief, participants were seated approximately 50–60 cm from the visual display (27” Apple iMac), which was presented in a 50 cm × 35 cm visual field. The screen brightness was set to maximum and all stimuli were presented within the center of the visual field. Individuals completed eight cycles consisting of 20 seconds eyes closed, followed by 40 seconds eyes open to a visual stimulus, consistent with prior NVC research (Phillips et al., 2015). Eight NVC cycles were completed by each participant to maximize the signal to noise ratio, as other physiological processes (e.g., Mayer Waves (Julien, 2006), respiratory sinus arrhythmia (Yasuma & Hayano, 2004), etc.) can cause variability within each cycle. A 40‐second eyes open protocol was utilized to ensure that peak PCA and MCA blood velocity were achieved, which usually occurs ~20 seconds following the stimulus presentation. Additionally, this was done to eliminate any human error associated with the stimulus presentation/withdrawal that may occur if only 30 seconds of data are collected.

### 2.3 Exercise protocols

All exercise was performed on a cycle ergometer (ergoline GmbH, Bitz, Germany). Each condition was completed following baseline measures. The HIIT protocol lasted 25 minutes and was broken down into a warm‐up phase, an exercise phase, and a cool‐down phase. A three‐minute warm‐up was followed by ten, one‐minute intervals of exercise at 85%–90% heart‐rate reserve (HRR) with one‐minute active rest periods in between each minute of work (Jung et al., 2015). The following Karvonen formula was used to calculate HRR (Miller et al., 1993):

$$\begin{array}{cc} \text{HRR} & =\text{Target Intensity}\left(\text{Age Predicted Maximum Heart Rate}‐\text{Resting Heart Rate}\right)\\ & \;+\text{Resting Heart Rate}). \end{array}$$

Participants were allowed three minutes of cool‐down after the intervals before starting recovery testing at hour zero. The MICT condition consisted of 45 minutes of exercise at ~50%–60% of HRR (Wewege et al., 2017). Participants sat quietly for 30 minutes in the control condition. These exercise protocols were selected because the MICT exercise intensity equates to ~60% VO_2max_, which would result in the highest hyperpneic‐induced elevations to CBV (MCA and PCA blood velocities); whereas HIIT represents ~85%–90% VO_2max_ in which CBV should be reduced from the peak level in MICT due to exercise‐induced hypocapnia (Marsden et al., 2012; Ogoh & Ainslie, 2009; Ogoh et al., 2005). Moreover, sporting events such as long‐distance cycling or distance running are typically represented by the MICT condition, whereas hockey or football events are more consistent with the HIIT condition. The former requires individuals to engage in long steady‐state bouts of exercise, compared to the latter, in which individuals perform short bouts of maximal exertion, followed by periods of intermittent rest/active recovery.

### 2.4 Data processing

**FIGURE 2:**
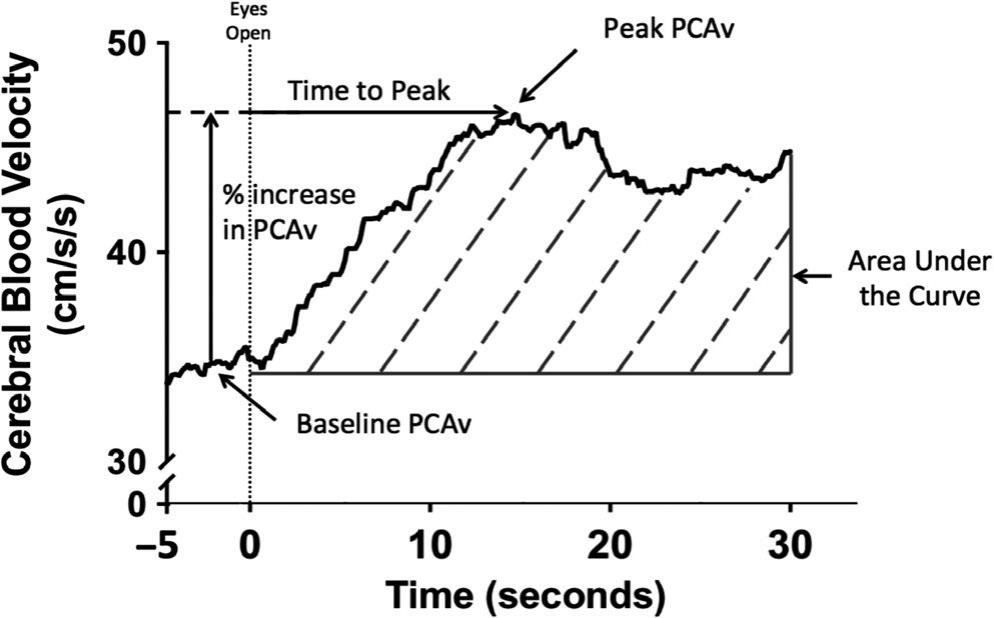
Representative average trace of the posterior cerebral artery (PCA) blood velocity from a participant at one time point (eight cycles) *during* a “*Where's Waldo”* visual scene search. Baseline PCA blood velocity was the average of the eyes closed (−5 to 0 seconds), peak PCA blood velocity was the maximal velocity during the eyes open visual search (0 to 30 seconds), percent (%) increase in PCA blood velocity was the relative increase from baseline to the peak PCA blood velocity, time‐to‐peak was the time from the eyes open stimulus to the peak PCA blood velocity, and the area‐under‐the‐curve during eyes open searching was calculated as the total activation during the first 30‐second eyes open, relative to the eyes‐closed baseline values. Centimeters per second per second (cm/s/s)

Using the R‐R intervals from the electrocardiogram, beat‐to‐beat heart rate, MAP, and mean PCA and MCA CBV were sampled. Breath‐to‐breath peak expired carbon dioxide values were extracted to measure P_ET_CO_2_. All signals were visually inspected for artifacts and data from each trial were aligned to stimulus onset (eyes open), and then averaged to generate one response per subject, per condition, per time point. The average of the 5 seconds preceding initiation (i.e., the last 5 seconds of eyes closed) is reported as the baseline CBV measure for each condition, whereas, the NVC responses were quantified during the 30 seconds after the “eyes‐open” command (Gommer et al., 2014; Smirl et al., 2016; Wright et al., 2017) (Figure 2). The NVC metrics were quantified within the PCA and MCA as area‐under‐the‐curve during the first 30 seconds after stimulus onset (AUC_30_), baseline CBV during the eyes closed section, peak CBV during the eyes open section, the time‐to‐peak CBV after stimulus onset, and the total percent increase in CBV (Figure 2). The AUC_30_ metric was derived based upon the baseline and peak CBV during each individual trial (i.e., total activation) (Smirl et al., 2016). This can be seen in Figure 2, where the baseline value from the last 5 seconds of the eyes closed section was used, and the total activation was calculated from the area change from baseline to peak CBV, over the 30 seconds of task stimulation (Smirl et al., 2016). Moreover, as each baseline CBV value was slightly different from trial‐to‐trial, calculating AUC_30_ this way minimizes the effect of trial‐to‐trial baseline variation. Data processing for all of the aforementioned variables was calculated in Excel (Microsoft, Redmond, Washington, United States).

### 2.5 Statistical analyses

#### 2.5.1 Sample size calculation

A previous study by Yamaguchi, Ikemura, Kashima, et al. (2015) was used to calculate the required sample size, as these authors examined the PCA blood velocity response to visual stimulation during rest compared to exercise at 120 bpm, 140 bpm, and 160 bpm. The sample size was calculated at a power of 0.80, with two‐tails, and an alpha of 0.05 between the control and exercise conditions regarding NVC metrics. It was determined nine participants were needed in each group to determine a statistical difference, which is analogous to published NVC research (Caldwell et al., 2018; Phillips et al., 2013, 2014; Willie, Cowan, et al., 2011). Moreover, a randomized cross‐over design was utilized so individuals could act as their own control, which reduces the likelihood confounding from unknown variables influences the results (i.e., concussion history, genetics, etc.) (Thiese, 2014).

#### 2.5.2 Effects of exercise on NVC

Statistical analyses were performed using SPSS version 25.0 and RStudio (v1.2.5042). A two‐factor repeated measures ANOVA, with levels consisting of condition (HIIT, MICT, control) and time (baseline, zero, one, two, four, six, and eight hour follow‐ups) was performed to determine the main effects. Bonferroni post‐hoc analyses were conducted to determine significant condition, time, and condition by time Greenhouse‐Geisser main effects. *A priori* Bonferroni corrected simple effect comparisons were performed between the follow‐up time points and the baseline data to establish when NVC measures were no longer different from the baseline data.

The within‐ and between‐day reliability was assessed through coefficient of variation (CoV), typical error of the measurement (TEM) (Hopkins, 2000), and intraclass correlation coefficients (ICC) (Koo & Li, 2016). Additionally, Bland‐Altman Plots with 95% limits of agreement were calculated to establish the between‐day reliability of these metrics, as this measure is more sensitive to variability within measures compared to ICC (Bland & Altman, 1986; Myles & Cui, 2007). The within‐subject CoV was calculated by taking the cerebrovascular, cardiovascular, and NVC data points from each participant and dividing the mean from the standard deviation (Tian, 2006). This was done to compare the CoV between baseline data prior to each intervention and across the control condition. The cut‐offs for CoV were characterized using a threshold of <20% as acceptable variation, in conjunction with similar physiological studies (Barnes et al., 2018; Burma et al., 2020d; Burma, Copeland, Macaulay, Khatra, Wright, et al., 2020; Dawson et al., 2015; Quan & Shih, 1996; Scott et al., 1989; Smirl et al., 2015). Moreover, the ICC estimate was utilized to determine if the within‐ and between‐day reliability was poor (<0.50), moderate (0.50–0.75), good, (0.75–0.90), or excellent (>0.90) (Koo & Li, 2016). Main effects are reported as: F‐value, p‐value, Power (P). Data are presented as mean ±SD. Significance was set a priori at *p* < 0.05 and corrected for multiple comparisons.

## 3 RESULTS

### 3.1 Cerebrovascular and cardiovascular parameters across the interventions and during exercise

Condition, time, and condition by time main effects for all cerebrovascular and cardiovascular variables are displayed in Table 1. At baseline, there were no differences in CBV metrics (MCA and PCA blood velocities) and P_ET_CO_2_ between all three conditions (all *p* > 0.557), which all had a very good CoV of <9% (Table 1). Likewise, baseline MAP and heart rate did not differ (all *p* > 0.405) and again demonstrated very good and good between‐day CoV of <7% and <15%, respectively (Table 1). During HIIT, P_ET_CO_2_ was lowered compared to control and MICT (*p* < 0.001), but MICT and control were not different (*p* = 0.225) (Table 1). There were no differences between control and HIIT in PCA blood velocity (*p* = 0.580) and MCA blood velocity (*p* = 0.252), however during the MICT exercise both PCA blood velocity (all *p* < 0.047) and MCA blood velocity (all *p* < 0.036) were increased relative to control and HIIT (Table 1). Finally, exercise caused elevations in MAP during HIIT in comparison to control (*p* = 0.004), but MICT (all *p* > 0.180) was not different from control or HIIT (Table 1). Conversely, both MICT (*p* < 0.001) and HIIT (*p* < 0.001) led to increased heart rate relative to the control resting condition, which was augmented further during HIIT compared to MICT (*p* < 0.001) (Table 1). Succeeding the HIIT intervention at time point zero, P_ET_CO_2_ and heart rate were reduced and elevated, respectively compared to the premeasures (all *p* < 0.001) (Table 1). However, MCA blood velocity, PCA blood velocity, and MAP were comparable to HIIT baseline by hour zero (all *p* > 0.157) (Table 1). At hour one, heart rate was still elevated compared to pre HIIT values (*p* = 0.048), but all other values were comparable to baseline (all *p* > 0.174) (Table 1). All HIIT hemodynamics were comparable to baseline from hour two onwards (Table 1). Following MICT, heart rate was the only metric elevated (*p* = 0.010) compared to baseline, which from hour one onwards, no hemodynamics were different from baseline (all *p* > 0.319) (Table 1). All hemodynamic parameters were comparable across the control condition at each time point (all *p* > 0.100) (Table 1).

**TABLE 1:** Cardiorespiratory and cerebrovascular variables in nine participants during all NVC assessments at baseline and following 30 minutes of control rest, 25 minutes of High‐Intensity Interval Training (HIIT), 45 minutes of Moderate‐Intensity Continuous Training (MICT). The main effects (condition, time, and condition x time) are displayed in a single column; however, each is associated with the physiological variable within the respective row

	Baseline	During	T0	T1	T2	T4	T6	T8	
Control									Condition effect
P_ET_CO_2_ (mmHg)	39 ± 3	38 ± 2	38 ± 2	38 ± 2	37 ± 3	40 ± 2	39 ± 3	38 ± 2	F_(2,192)_ = 29.2, *p* < 0.001
RR (BPM)	15 ± 2	16 ± 3	16 ± 2	16 ± 3	16 ± 3	16 ± 3	16 ± 2	16 ± 2	F_(2,192)_ = 2.36, *p* = 0.098
MCAbv (cm/s)	65 ± 8	63 ± 7	63 ± 8	64 ± 7	63 ± 9	64 ± 8	62 ± 6	62 ± 8	F_(2,192)_ = 11.4, *p* < 0.001
PCAbv (cm/s)	38 ± 4	38 ± 6	41 ± 7	41 ± 6	42 ± 6	40 ± 6	39 ± 6	40 ± 5	F_(2,192)_ = 4.94, *p* = 0.008
MAP (mmHg)	87 ± 5	95 ± 10	90 ± 10	97 ± 13	96 ± 11	87 ± 15	93 ± 8	97 ± 8	F_(2,192)_ = 0.62, *p* = 0.539
HR (bpm)	68 ± 8	68 ± 8	63 ± 8	62 ± 10	62 ± 10	73 ± 9	66 ± 10	63 ± 9	F_(2,192)_ = 65.7, *p* < 0.001
HIIT									Time Effect
P_ET_CO_2_ (mmHg)	39 ± 3	31 ± 4^*†^	28 ± 4^*†^	37 ± 2	37 ± 2	39 ± 2	38 ± 2	38 ± 2	F_(7,192)_ = 15.0, *p* < 0.001
RR (bpm)	15 ± 3	41 ± 6^*†^	23 ± 4^*†^	16 ± 3	16 ± 3	16 ± 3	15 ± 3	16 ± 3	F_(7,192)_ = 4.00, *p* < 0.001
MCAbv (cm/s)	60 ± 8	58 ± 11	54 ± 8	57 ± 9	56 ± 8	57 ± 8	56 ± 6	57 ± 5	F_(7,192)_ = 0.19, *p* = 0.988
PCAbv (cm/s)	42 ± 7	36 ± 5	37 ± 5	38 ± 7	38 ± 8	40 ± 8	40 ± 7	40 ± 6	F_(7,192)_ = 0.16, *p* = 0.993
MAP (mmHg)	89 ± 7	113 ± 14^*†^	91 ± 16	97 ± 7	94 ± 7	94 ± 8	91 ± 9	94 ± 10	F_(7,192)_ = 2.43, *p* = 0.021
HR (bpm)	70 ± 10	167 ± 8^*†^	106 ± 10	81 ± 9^†^	74 ± 9	79 ± 10	73 ± 10	69 ± 10	F_(7,192)_ = 17.9, *p* < 0.001
MICT									Condition × Time Effect
P_ET_CO_2_ (mmHg)	38 ± 2	40 ± 4^‡^	37 ± 2^‡^	37 ± 3	37 ± 2	39 ± 3	39 ± 2	38 ± 2	F_(14,192)_ = 9.67, *p* < 0.001
RR (bpm)	15 ± 3	26 ± 5^*†‡^	16 ± 3^‡^	15 ± 2	15 ± 2	16 ± 3	16 ± 3	15 ± 3	F_(14,192)_ = 2.88, *p* = 0.001
MCAbv (cm/s)	61 ± 10	69 ± 6^*†‡^	63 ± 10^‡^	60 ± 11	61 ± 12	60 ± 9	61 ± 10	61 ± 11	F_(14,192)_ = 0.32, *p* = 0.991
PCAbv (cm/s)	41 ± 8	43 ± 4^*†‡^	43 ± 5^‡^	41 ± 7	42 ± 7	43 ± 8	43 ± 8	44 ± 7	F_(14,192)_ = 0.57, *p* = 0.885
MAP (mmHg)	90 ± 8	109 ± 20	92 ± 10	99 ± 7	95 ± 12	95 ± 9	94 ± 10	94 ± 10	F_(14,192)_ = 0.17, *p* = 0.999
HR (bpm)	68 ± 8	136 ± 4^*†‡^	83 ± 9^†‡^	67 ± 4^‡^	64 ± 9	71 ± 8	65 ± 10	63 ± 7	F_(14,192)_ = 7.55, *p* < 0.001

Values are means ±standard deviation. End‐tidal values of carbon dioxide (P_ET_CO_2_), respiratory rate (RR), breaths per minute (BPM) millimeters of mercury (mmHg), middle cerebral artery blood velocity (MCAv), centimeters per second (cm/s), posterior cerebral artery blood velocity (PCAv), mean arterial pressure (MAP), beats per minute (bpm). The asterisk (*) denotes a value that is different from its own respective Pre value at *p* < 0.05. The dagger (†) denotes a value that is different from the control condition at each respective time point at *p* < 0.05. The diesis (‡) denotes a value that is different from the HIIT condition at each respective time point at *p* < 0.05).

### 3.2 Condition, time, and condition by time main effects in the PCA

**FIGURE 3:**
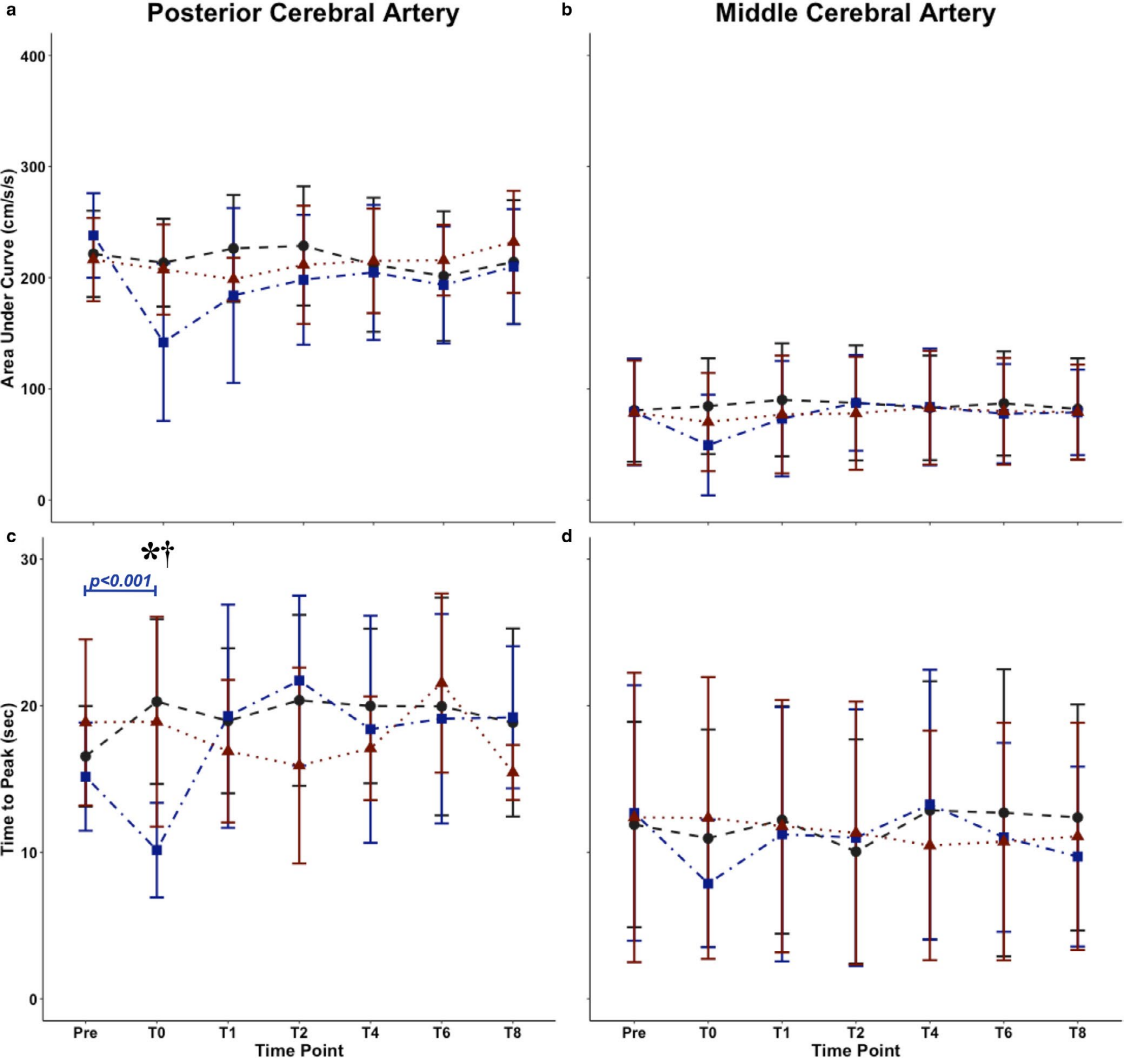
Mean with standard deviation of area‐under‐the‐curve (AUC_30_) in the (A) posterior cerebral artery (PCA) and (B) middle cerebral artery (MCA) and the time‐to‐peak in the (C) PCA and (D) MCA across the day in nine individuals. These measures were calculated during the visual task. Main effects for PCA AUC_30_: condition (F_(2,25)_ = 3.11, *p* = 0.047, *p* = 0.99), time (F_(6,84)_ = 1.54, *p* = 0.169, *p* = 0.99), and condition by time (F_(12,177)_ = 1.02, *p* = 0.434, *p* = 0.84). Main effects for MCA AUC_30_: condition (F_(2,25)_ = 0.66, *p* = 0.521, *p* = 0.82), time (F_(6,84)_ = 0.35, *p* = 0.911, *p* = 0.18), and condition by time (F_(12,177)_ = 0.19, *p* = 0.999, *p* = 0.07). Main effects for time‐to‐peak PCA blood velocity: condition (F_(2,25)_ = 1.66, *p* = 0.194;*p* = 0.99), time (F_(6,84)_ = 1.45, *p* = 0.200, *p* = 0.99); and condition by time (F_(12,177)_ = 2.15, *p* = 0.016, *p* = 0.99). Main effects for time‐to‐peak MCA blood velocity: condition (F_(2,25)_ = 0.19, *p* = 0.828, *p* = 0.12), time (F_(6,84)_ = 0.21, *p* = 0.973, *p* = 0.09), and condition by time (F_(12,177)_ =0.22, *p* = 0.997, *p* = 0.08). The asterisk (*) denotes HIIT varied from control at hour zero in time‐to‐peak PCA measures (*p* = 0.002). The dagger (†) denotes HIIT and moderate‐intensity continuous training (MICT) were different at hour zero (*p* = 0.008). The control condition is shown in gray (dashed), MICT in red (dotted), and HIIT in blue (dashed‐dotted)

**FIGURE 4:**
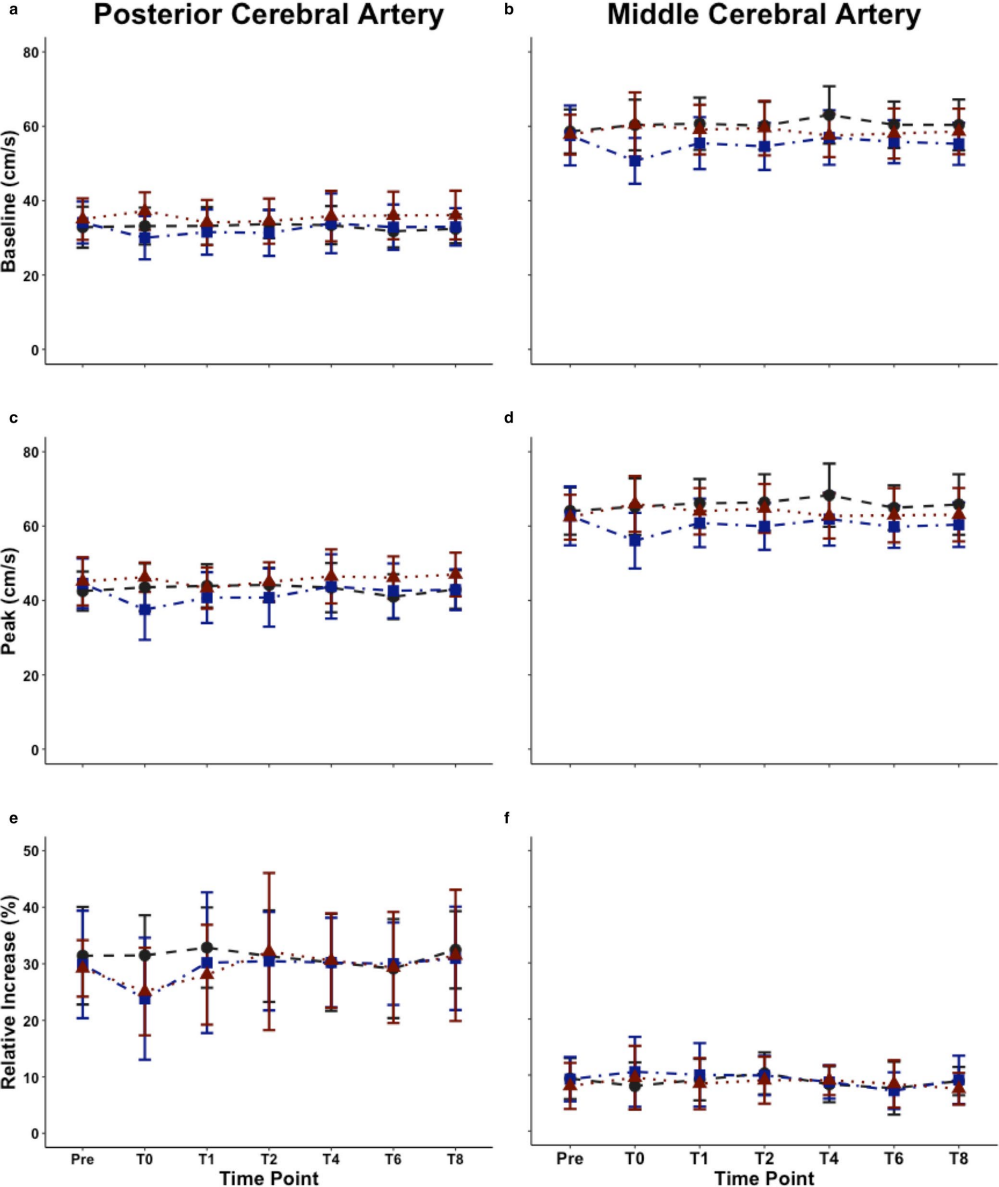
Mean ± standard deviation values of A) baseline posterior cerebral artery (PCA) blood velocity, B) baseline middle cerebral artery (MCA) blood velocity, C) peak PCA blood velocity, D) peak MCA blood velocity, E) the relative percent (%) increase in PCA blood velocity from the eyes closed to eyes open stimulus, and F) the relative percent (%) increase in MCA blood velocity from the eyes closed to eyes open stimulus across the day in nine individuals. Baseline measures were calculated during the 5 seconds preceding the eyes open stimulation, whereas peak and the % increase were calculated during the visual task. Main effects for baseline PCA blood velocity: condition (F_(2,25)_ = 5.50, *p* = 0.005, *p* = 0.99), time (F_(6,84)_ = 0.198, *p* = 0.977, *p* = 0.09), and condition by time (F_(12,177)_ = 0.420, *p* = 0.954, *p* = 0.17). Main effects for baseline MCA blood velocity: condition (F_(2,25)_ = 10.7, *p* < 0.001, *p* = 0.99), time (F_(6,84)_ = 0.23, *p* = 0.967, *p* = 0.09), and condition by time (F_(12,177)_ = 0.68, *p* = 0.770, *p* = 0.42). Main effects for peak PCA blood velocity: condition (F_(12,177)_ = 5.77, *p* = 0.004, *p* = 0.99), time (F_(2,25)_ = 0.44, *p* = 0.85, *p* = 0.28), and condition by time (F_(6,84)_ = 0.70, *p* = 0.75, *p* = 0.46). Main effects for peak MCA blood velocity: condition (F_(2,25)_ = 10.5, *p* < 0.001, *p* = 0.99), time (F_(6,84)_ =0 .25, *p* = 0.960, *p* = 0.11), and condition by time (F_(12,177)_ = 0.62, *p* = 0.823, *p* = 0.36). Main effects for the relative percent (%) increase in PCA blood velocity: condition (F_(2,25)_ = 0.91, *p* = 0.403, *p* = 0.98) time (F_(6,84)_ = 0.83, *p* = 0.23, *p* = 0.83), and condition by time (F_(12,177)_ = 0.31, *p* = 0.26, *p* = 0.11). Main effects for the relative percent (%) increase in MCA blood velocity: condition (F_(2,25)_ = 0.44, *p* = 0.644, *p* = 0.47), time (F_(6,84)_ = 0.68, *p* = 0.667, *p* = 0.63), or condition by time (F_(12,177)_ = 0.318, *p* = 0.985, *p* = 0.11). The control, moderate‐intensity continuous training (MICT), and high‐intensity interval training (HIIT) traces are displayed in gray (dashed), blue (dashed‐dotted), and red (dotted), respectively. Centimeters per second (cm/s)

For the total AUC_30_ within the PCA, a condition main effect was found (F_(2,25)_ = 3.43, *p = *0.035, *p* = 0.99); however, there were no time (F_(6,84)_ = 1.71, *p* = 0.121, *p* = 0.99) or condition by time interaction main effects (F_(12,177)_ = 1.15, *p* = 0.324, *p* = 0.93) (Figure 3A). Within time‐to‐peak PCA blood velocity there were no condition (F_(2,25)_ = 1.66, *p* = 0.194; *p* = 0.99) or time main effects (F_(6,84)_ = 1.45, *p* = 0.200, *p* = 0.99); however a condition by time interaction main effect was noted (F_(12,177)_ = 2.15, *p* = 0.016, *p* = 0.99) (Figure 3C). No time (F_(6,84)_ = 0.198, *p* = 0.977, *p* = 0.09) or condition by time interaction main effects (F_(12,177)_ = 0.420, *p* = 0.954, *p* = 0.17) were found within baseline PCA blood velocity metrics; however, a condition main effect was present (F_(2,25)_ = 5.50, *p* = 0.005, *p* = 0.99) (Figure 4A). Only a condition main effect was present within peak PCA blood velocities measures (F_(2,25)_ = 5.77, *p* = 0.004, *p* = 0.99) as time (F_(6,84)_ = 0.44, *p* = 0.85, *p* = 0.28) and condition by time (F_(12,177)_ = 0.70, *p* = 0.75, *p* = 0.46) main effects were not significant (Figure 4C). Finally, regarding the percent increase in PCA blood velocity, condition (F_(2,25)_ = 0.91, *p* = 0.403, *p* = 0.98), time (F_(6,84)_ = 0.83, *p* = 0.23, *p* = 0.83), and condition by time (F_(12,177)_ = 0.31, *p* = 0.26, *p* = 0.11) were not significant (Figure 4E).

### 3.3 Condition, time, and condition by time main effects in the MCA

No condition (F_(2,25)_ = 0.66, *p* = 0.521, *p* = 0.82), time (F_(6,84)_ = 0.35, *p* = 0.911, *p* = 0.18), or condition by time (F_(12,177)_ = 0.19, *p* = 0.999, *p* = 0.07) main effect was found for AUC_30_ within the MCA (Figure 3B). Similarly, no main effects were present for condition (F_(2,25)_ = 0.19, *p* = 0.828, *p* = 0.12), time (F_(6,84)_ = 0.21, *p* = 0.973, *p* = 0.09), and condition by time (F_(12,177)_ = 0.22, *p* = 0.997, *p* = 0.08) regarding time‐to‐peak MCA blood velocity metrics (Figure 3D). A condition main effect was found within baseline MCA blood velocity metrics (F_(2,25)_ = 10.7, *p* < 0.001, *p* = 0.99), however no time (F_(6,84)_ = 0.23, *p* = 0.967, *p* = 0.09) or condition by time main effect was found (F_(12,177)_ = 0.68, *p* = 0.770, *p* = 0.42) (Figure 4B). Likewise, within peak MCA blood velocity metrics, no time (F_(6,84)_ = 0.25, *p* = 0.960, *p* = 0.11) or condition by time main effect was found (F_(12,177)_ = 0.62, *p* = 0.823, *p* = 0.36); however a condition main effect was found (F_(2,25)_ = 10.5, *p* < 0.001, *p* = 0.99) (Figure 4D). No condition (F_(2,25)_ = 0.44, *p* = 0.644, *p* = 0.47), time (F_(6,84)_ = 0.68, *p* = 0.667, *p* = 0.63), or condition by time (F_(12,177)_ = 0.318, *p* = 0.985, *p* = 0.11) main effect was found for the percent increase in MCA blood velocity (Figure 4F).

### 3.4 Neurovascular coupling data from pre‐post exercise conditions

The post hoc comparisons revealed AUC_30_ was attenuated across HIIT compared to the control condition (mean difference of −23 cm/s/s) (*p* = 0.045) (Figure 3A). This difference between conditions was largely attributable to the difference at hour zero (−77 cm/s/s) (Figure 3A). The mean difference at the other time points was: baseline (17 cm/s/s), hour one (−42 cm/s/s), hour two (−30 cm/s/s), hour four (−7 cm/s/s), hour six (−8 cm/s/s), and hour eight (−4 cm/s/s) (Figure 3A). The post hoc comparison for the time‐to‐peak PCA blood velocity interaction term, demonstrated this was reduced following HIIT at hour zero compared with control (*p* = 0.002) and MICT (*p* = 0.008) at hour zero (Figure 3C). Time‐to‐peak PCA blood velocity was consistent between MICT baseline and hour zero (*p* = 0.990) and between MICT and control at hour zero (*p* = 0.861) (Figure 3C). From hour one and onwards during the HIIT condition, time‐to‐peak PCA blood velocity did not differ from baseline (all *p* > 0.131) (Figure 3C).

The condition main effect for baseline PCA blood velocity demonstrated MICT was greater than both control (mean difference = 2.6 cm/s) (*p* = 0.030) and HIIT (mean difference = 3.2) (*p* = 0.006) (Figure 4A). This difference was largely influenced at hour zero, where baseline PCA blood velocity increased to 4 cm/s and 7 cm/s during MICT compared to control and HIIT, respectively (Figure 4A). Conversely, all of the other mean differences at each time point were 2 (control) and ≤3 cm/s/s (HIIT) (Figure 4A). Likewise, peak PCA blood velocity followed a similar trend across the day and was dissimilar between HIIT and MICT (mean difference =3.8 cm/s) (*p* = 0.003) and HIIT and control at hour zero (mean difference = 2.5 cm/s) (*p* = 0.046) (Figure 4C).

For baseline MCA blood velocity, the post hoc comparisons revealed a difference between HIIT and control (mean difference = −5.3 cm/s) (*p* < 0.001) and HIIT and MICT (mean difference = −3.5 cm/s) (*p* < 0.010) (Figure 4B). Similarly, a difference was present between HIIT and control (mean difference = −5.6 cm/s) (*p* < 0.001) and HIIT and MICT (mean difference = −3.4 cm/s) (*p* = 0.016) (Figure 4D).

### 3.5 Within‐ and between‐day reliability of neurovascular coupling metrics

**FIGURE 5:**
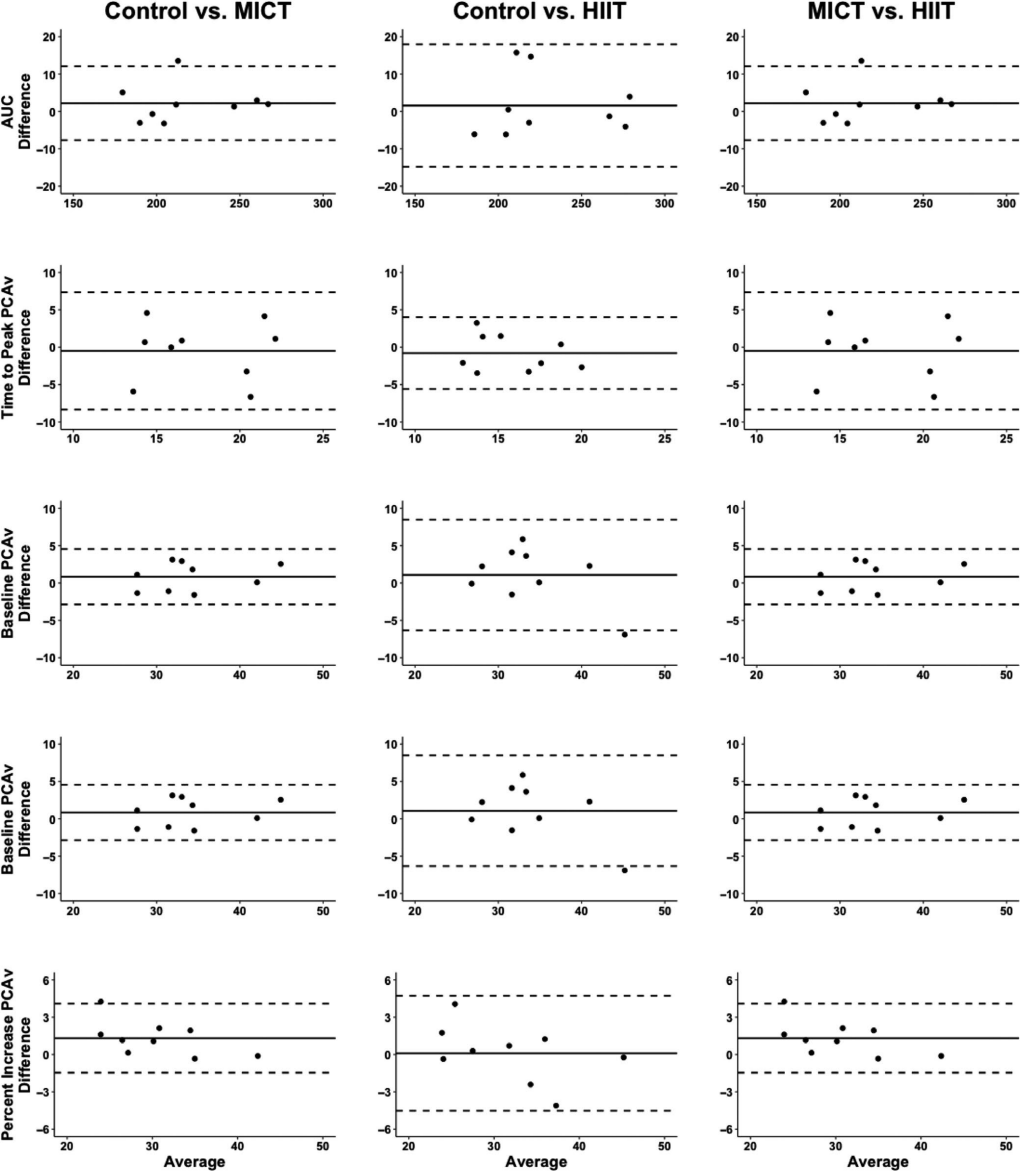
Bland‐Altman Plot with 95% limits of agreement demonstrating the *between*‐*day reliability* of the neurovascular coupling response within the posterior cerebral artery between the three conditions (control, moderate‐intensity continuous training [MICT], and high‐intensity interval condition [HIIT]) in nine individuals. Area‐under‐the‐curve (AUC) and posterior cerebral artery blood velocity (PCAv)

**FIGURE 6:**
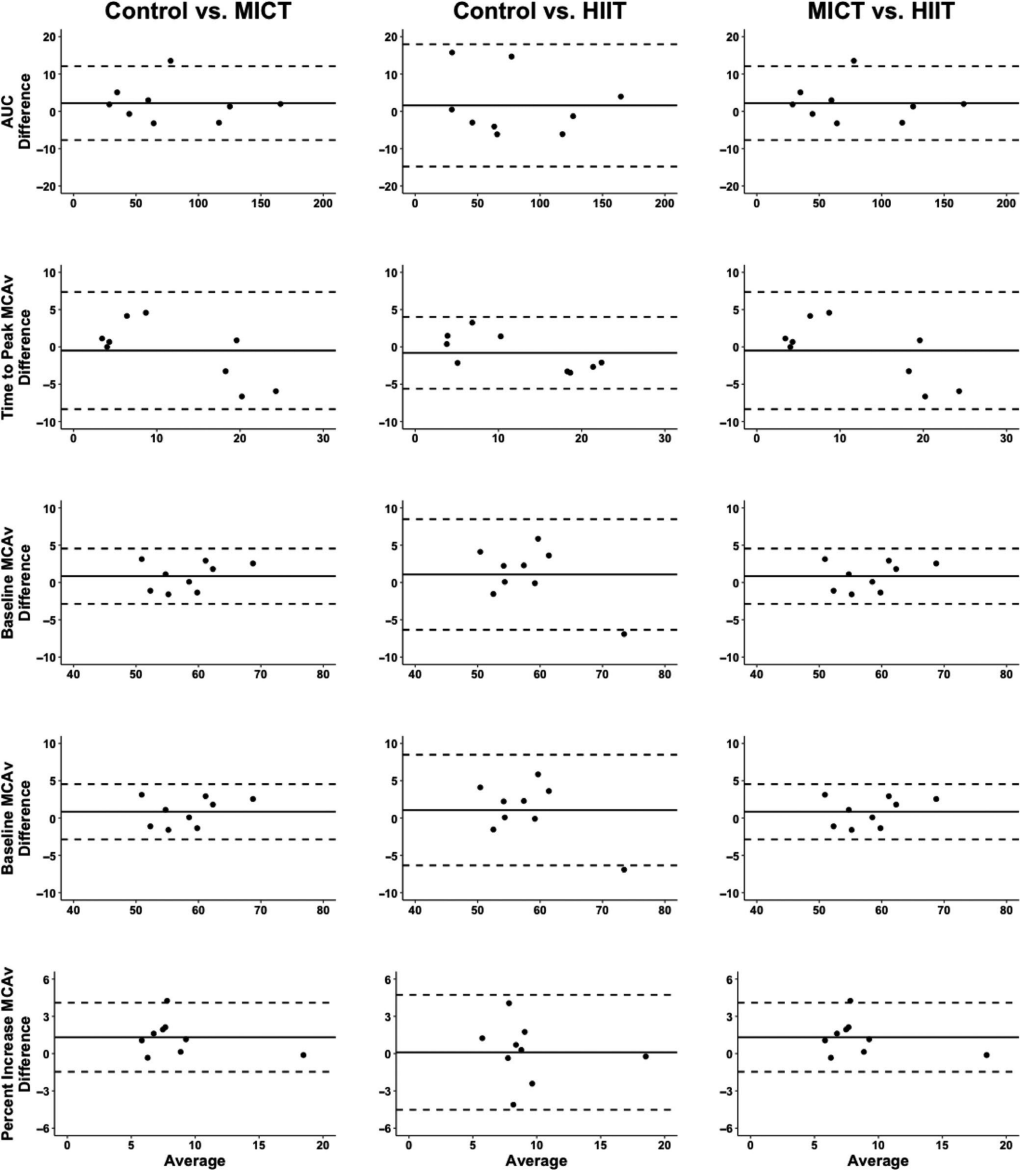
Bland‐Altman Plot with 95% limits of agreement demonstrating the *between*‐*day reliability* of the neurovascular coupling response within the middle cerebral artery between the three conditions (control, moderate‐intensity continuous training [MICT], and high‐intensity interval condition [HIIT]) in nine individuals. Area‐under‐the‐curve (AUC) and middle cerebral artery blood velocity (MCAv)

Table 2 displays that the within‐ and between‐day variability (CoV), was excellent within both baseline and peak PCA and MCA blood velocity measures. Likewise, for the most part, AUC_30_ and percent increase displayed acceptable levels of variability; however, the one exception was the percent increase in MCA blood velocity across the control day (CoV = 25%) (Table 2). Additionally, the within‐day time‐to‐peak PCA and MCA blood velocity metrics were greater than the *a priori* established threshold of 20% (Table 2). The vast majority of NVC metrics displayed excellent levels of reliability (>0.90), when expressed through ICC (Table 2). However, time‐to‐peak PCA blood velocity displayed poor within‐day and between‐day reliability (<0.50); whereas the ICC was good (0.75–0.90) in the MCA for time‐to‐peak metrics (Table 2). Relative to the mean for each value, the TEM measures displayed little variance in all NVC metrics (Table 2). A similar trend was noted for the Bland‐Altman Plots with 95% limits of agreement when measuring between baseline comparisons, where relative to the mean they all displayed good levels of reliability, aside from time‐to‐peak CBV metrics (Figures 5 and 6).

**TABLE 2:** The within‐ and between‐day reliability of neurovascular coupling metrics within the posterior and middle cerebral arteries during a complex, visual scene search task (“*Where's Waldo*”) in nine individuals. Reliability was measured through coefficient of variation (CoV), typical error of the measurement (TEM), and intraclass correlation coefficient (ICC) metrics

	Posterior Cerebral Artery				
	AUC_30_	Time‐to‐Peak	Baseline	Peak	Percent Increase
CoV (%)					
Within‐Day	11.3	22.5	5.2	5.4	11.2
Between‐Day	8.6	17.8	3.3	4.3	7.4
TEM					
Within‐Day	4.9	2.2	1.4	1.6	1.8
Between‐Day	4.5	2.1	1.2	1.3	2.0
ICC					
Within‐Day	0.949	0.492	0.982	0.970	0.966
Between‐Day	0.794	0.387	0.979	0.966	0.871

	Middle Cerebral Artery				
	AUC_30_	Time‐to‐Peak	Baseline	Peak	Percent Increase
CoV (%)					
Within‐Day	10.5	23.0	4.3	4.3	25.0
Between‐Day	5.4	15.6	2.5	2.2	13.7
TEM					
Within‐Day	3.2	2.2	1.6	1.6	1.4
Between‐Day	2.7	1.3	1.5	1.3	1.2
ICC					
Within‐Day	0.993	0.789	0.969	0.969	0.928
Between‐Day	0.997	0.759	0.957	0.969	0.942

Area‐under‐the‐curve during the first 30 seconds of presentation to the visual stimuli (AUC_30_).

## 4 DISCUSSION

This was the first study to empirically examine the time‐course recovery that acute HIIT and MICT has on NVC metrics. The key findings from this investigation were: 1) the NVC response was attenuated following HIIT at T0 but remained stable following MICT; 2) the alterations in NVC following HIIT recovered 1 hour post exercise; 3) both HIIT and MICT impacted baseline and peak CBV within the first 10 minutes following exercise, which fully recovery by 1 hour post exercise; and 4) NVC parameters demonstrated a high degree of within‐and between‐day reproducibility. Collectively, these results demonstrate NVC measures are impacted by high‐intensity exercise above anaerobic threshold. Therefore the previously recommended four‐ to twenty‐four hour time restriction for research investigations is conservative as the current data reveal these measures have returned to baseline just one hour following an acute bout of high‐intensity exercise. However, as exercise, irrespective of intensity, results in alterations to numerous physiological systems (e.g., cardiovascular, cerebrovascular, respiratory, etc.) it is important baseline physiological values have been restored prior to NVC assessments. Finally, NVC metrics demonstrated low levels of variability and a high degree of within‐ and between‐day reliability of these measures between the hours of 8:00am and 7:00 pm.

### 4.1 Comparison with previous studies

This investigation expands upon the previous findings by Willie, Cowan, et al. (2011) as NVC was examined throughout the recovery period, however like the aforementioned study, no changes were found for NVC metrics during acute recovery following MICT (Figures 3 and 4). Conversely, Yamaguchi, Ikemura, Kashima, et al. (2015) demonstrated exercise at moderate (140 bpm) and heavy (160 bpm) intensities increased PCA blood velocity during the eyes‐closed period preceding the visual stimulation task. Furthermore, they found the eyes‐closed baseline and peak PCA blood velocity were increased during all exercise conditions (Yamaguchi, Ikemura, Kashima, et al., 2015). Similarly, while no differences were noted relative to baseline, this study showed that for the comparisons between conditions at T0, a condition main effect was present for both baseline and peak blood velocity within the PCA and MCA (Figure 4). This is likely attributable to the fact that exercise at moderate intensities is known to result in hyperpnea‐induced vasodilation; whereas, exercise at intensities above anaerobic threshold induces hyperventilation‐induced vasoconstriction (Smith & Ainslie, 2017). These results are consistent with previous literature demonstrating alterations to CBV within the first few minutes following exercise (Smith et al., 2014). Furthermore, Yamaguchi, Ikemura, & Hayashi (2015) examined the NVC response during exercise to exhaustion and in the immediate initial recovery (three minutes) following exercise. They demonstrated a blunt increase in PCA blood velocity response to visual stimulation (NVC) at the exhaustive exercise level as compared to baseline; however, this response then returned to preexercise levels during the immediate three‐minute recovery period (Yamaguchi, Ikemura, & Hayashi, 2015). Analogously, there were no differences within this investigation regarding the percent increase in PCA blood velocity during acute recovery (0–8 hours) (Figure 4E).

### 4.2 Potential mechanisms underlying the alterations between exercise conditions

Individuals are able to engage in exercise for extended durations, as these intensities are below a threshold of ~60–70% VO_2max_ (Mateika & Duffin, 1995). However, it is important to note this is slightly different for all individuals. Nonetheless, during exercise at or slightly below this threshold, there will be a relatively linear increase in both oxygen consumption and carbon dioxide production (Mateika & Duffin, 1995). This is the resultant as the body will primarily be utilizing aerobic glycolysis to produce adenosine triphosphate (ATP) (Swanwick, 2018). In turn, this elevation in circulating carbon dioxide molecules will cause a vasodilatory response within the cerebrovasculature (Marsden et al., 2012; Ogoh & Ainslie, 2009; Smith & Ainslie, 2017). Therefore, at exercise intensities similar to that of the MICT condition used in this investigation, CBV will be elevated due to hyperpnic‐induced vasodilation (Marsden et al., 2012; Ogoh & Ainslie, 2009; Smith & Ainslie, 2017). Table 1 highlights the MICT condition achieved this desired response as compared to control, P_ET_CO_2_ values was slightly increased from 38 mmHg to 40 mmHg. More so, MCA and PCA blood velocities increased from both their baseline as well as from the control values, further providing support that hyperpnic‐induced vasodilation occurred.

However, exercise intensities above this threshold, cause the body to switch from aerobic to anaerobic glycolysis, where ATP production primarily comes from glucose and the phosphocreatine system (Beaver et al., 1986; Swanwick, 2018). At this point, carbon dioxide production is augmented compared to oxygen utilization, resulting in the respiratory exchange ratio to exceed 1.00 (Smith & Ainslie, 2017). Additionally, the elevated carbon dioxide and associated hydrogen ions, will increase the acidity of the cerebrovasculature, which stimulates the chemoreceptors to increase respiration (Beaver et al., 1986). Through the bicarbonate buffering system, the surge in carbon dioxide molecules will be expelled in an attempt for the cardiovascular system to restore homeostasis, allowing for an individual to continue exercising (Beaver et al., 1986). The increased respiratory response results in reductions in the concentration of arterial carbon dioxide, which in turn leads to the cerebrovasculature to constrict (Marsden et al., 2012; Ogoh & Ainslie, 2009; Smith & Ainslie, 2017). This response to high‐intensity exercise is also known as hyperventilation‐induced vasoconstriction (Marsden et al., 2012; Ogoh & Ainslie, 2009; Smith & Ainslie, 2017). Table 1 provides support this physiological response occurred, where P_ET_CO_2_ values were reduced compared to both MICT, control, and its own baseline values, while CBV was similar or slightly lower during the HIIT protocol. Moreover, the physiological results from the current exercise modalities are homogeneous to a previous study by Smith and colleagues (Smith et al., 2014) who determined the same physiological parameters, based upon an individual's maximal wattage. Therefore, while a limitation of this investigation is that each individual's precise VO_2max_ was not calculated, the physiological responses that occurred were indicative of the participants being below (MICT) and above (HIIT) anaerobic threshold, respectively.

The 40% decrease in total activation (AUC_30_) and 33% reduction in time‐to‐peak PCA blood velocity activation immediately following the HIIT condition can likely be attributed to extended effects associated with hyperventilation‐induced reductions in PCA blood velocity (Figure 3). For example, a previous report revealed the absolute and relative peak CBV response were slower, while the total activation and the time‐to‐peak CBV were blunted when comparing NVC responses between normo‐ and hyper‐ventilatory conditions (Szabo et al., 2011). This is directly congruent with the results in this investigation at time zero, where P_ET_CO_2_ was reduced immediately following HIIT (28 ± 4 mmHg) compared to HIIT baseline (39 ± 3 mmHg) and control at hour zero (38 ± 2 m mmHg) (Table 1). Likewise, respiratory rate was increased following HIIT (23 ± 4 breaths per minute [BPM]) compared to MICT (16 ± 3 BPM) and the control condition (16 ± 2 BPM) (Table 1). Therefore, it appears that the ventilatory alterations associated with HIIT exercise, caused a decreased compliance of the cerebrovasculature, explaining the alterations in AUC_30_ and time‐to‐peak PCA blood velocity (Figure 3A,C). However, it should be noted that the time‐to‐peak metrics displayed the greatest amount of variability within both the PCA and MCA. Thus, it cannot be discredited that the changes in the PCA regarding time‐to‐peak values contained some degree of measurement error. Nevertheless, all nine participants experienced a reduction in their time‐to‐peak metric compared to baseline and control values. As well, as described above, there is plausible physiological evidence to explain this phenomenon, and therefore it appears this change can be attributable to high‐intensity exercise. These results were further confirmed by Yamaguchi, Ikemura, & Hayashi (2015) who found exhaustive exercise reduced P_a_CO_2_ 7 ± 2%, resulting in diminished NVC responses. Moreover, Yamaguchi et al. (2014) demonstrated the NVC response is maintained when participants performed a static handgrip exercise, which resulted in an acute elevation to MAP. Although MAP was elevated during HIIT, it returned to baseline at hour zero following and thus likely did not contribute to the attenuated NVC response following high‐intensity exercise.

Exercise is also known to augment sympathetic nerve activity (i.e., elevated release of catecholamines, cortisol, and other circulating hormones (Brenner et al., 1997)) through sympathetic activation and parasympathetic withdrawal (Stanley et al., 2013). With the termination of exercise, parasympathetic reactivation occurs (Burma et al., 2020a; Heffernan et al., 2006; Seiler et al., 2007; Terziotti et al., 2001), which has shown to be impacted by the quantity of stress metabolites circulating the cerebrovasculature (Stanley et al., 2013). Nonetheless, previous research has suggested that the influences of autoregulation and cerebrovascular reactivity mask the subtle changes to sympathetic nerve activity (Ainslie & Duffin, 2009; Levine & Zhang, 2008; Lieshout & Secher, 2008; Strandgaard & Sigurdsson, 2008). This supports the notion that ventilatory variables should be controlled for during future NVC assessments. Moreover, several studies have highlighted that a complex interaction occurs between changes in P_ET_CO_2_ values (i.e., hypercapnia and hypocapnia) and the NVC response (Maggio et al., 2013, 2014; Szabo et al., 2011). Therefore, future investigations are warranted to elucidate how the findings may change when participants breathe at eucapnia immediately following HIIT.

### 4.3 Between‐day and within‐day variability of neurovascular coupling metrics

This was the first study to assess the within‐ and between‐day variability of NVC metrics at seven and three time points, respectively. It has been demonstrated that between‐day measures of CBV are relatively stable with several studies showing baseline testing variations of <5%, with the minor changes between days likely occurring due to biological factors (Smirl et al., 2015). As NVC parameters are derived from CBV metrics, it is understandable why they exhibited excellent levels of between‐day variation at baseline (Table 2). However, time‐to‐peak metrics within both the PCA and MCA was the one exception, which had between‐day ICC of 0.39 and 0.76, respectively. Additionally, the Bland‐Altman Plots with 95% limits of agreement were centered near a mean difference of 0, with narrow limits of agreement for NVC parameters (Figures 5 and 6). Homogenously, the control data set in the current investigation revealed minimal alterations to the NVC response across the day (Table 2), with excellent ICC (i.e., >0.90), aside from time‐to‐peak metrics. Interestingly, the percent increase within the MCA displayed the greatest CoV (Table 2). This is likely attributable to the fact the NVC response utilized in the present investigation (“*Where's Waldo)* elicits a more robust response within PCA blood velocity than MCA blood velocity.

Typically, it has been reported that a 10%–20% increase in CBV occurs during a NVC task (Phillips et al., 2015). Contrarily, in this investigation, the percent increase in PCA blood velocity was 20%–30% in most individuals (Figure 4E). The differences between studies is the resultant that a complex, visual paradigm used in this study (Smirl et al., 2016), which elicits a larger response compared to reading or other visual tasks that the typical 10%–20% increase was derived from (Phillips et al., 2015). Finally, the percent increase in the MCA blood velocity during the visual task is directly comparable (i.e., 5%–8%) to the typical response seen during a visual task (Phillips et al., 2015) (Figure 4F). Additionally, the right MCA was insonated on purpose, given that the right hemisphere of the brain is activated to a great extent than the left hemisphere during visuo‐spatial tasks (Corballis, 2003). Therefore, a slightly stronger NVC response should theoretically occur within the right MCA compared to the left. Further, while the post hoc power calculations were typically lower within the MCA compared to the PCA, this again is relatable to the stimulus utilized as it is known to elicit a more robust response within the PCA compared to the MCA (Smirl et al., 2016). Collectively, future studies examining the NVC response using the complex scene search paradigm “*Where's Waldo*” can reliably assess NVC metrics at any time point between 8:00am and 7:00 pm.

### 4.4 Implications for future neurovascular coupling assessments

There are several implications the current findings have for future studies examining NVC metrics. The present data will give researchers an evidence‐based time restraint of one hour during which participants need to refrain from exercise to ensure the history of exercise will have a minimal influence on their data. It should also be noted although the suggested time restraint of one hour is best applied to more intense forms of exercise, a conservative approach would be to apply this to all forms of exercise. Additionally, while the NVC metrics were comparable to resting values, the participant's heart rates were still slightly elevated. Therefore, prior to data collection, researchers should ensure that a participant's resting hemodynamic represents typically seen values. Further, this study increases the ability to perform NVC response investigations with populations of active individuals or sports teams, who perform games and/or training sessions daily. Finally, as no changes were found across the control day, the influence of diurnal variation on NVC measures appears to be minimal.

### 4.5 Limitations

An important limitation of transcranial Doppler ultrasound is its inability to directly measure flow; however, it is able to index CBV based on the assumption that velocity is equivalent for flow as long as the vessel being insonated does not change (Ainslie & Hoiland, 2014). Studies examining cerebral vessels using high‐resolution magnetic resonance imaging have demonstrated the diameter of cerebral arteries is relatively constant when P_ET_CO_2_ is within eight mmHg of eucapnia (35–45 mmHg) (Coverdale et al., 2014; Verbree et al., 2014). Therefore, regardless of this limitation due to the use of TCD, our data can be reliably portrayed as an accurate representative index of CBV. Another limitation is due to the small sample size of nine participants. However, employing a randomized cross‐over study design within the same cohort of individuals enables participants to act as their own controls, which in turn minimizes the likelihood of covariates influencing the present findings (Thiese, 2014). Further, the MICT and HIIT exercise intensities were not individually calculated for each participant, but rather were based upon the formula for maximal HR (220‐age) (Miller et al., 1993). Nonetheless, even though each participant's precise fitness and VO_2max_ were not determined, they all appeared to be working below the anaerobic threshold during MICT and above anerobic threshold during HIIT. Additionally, these intensities were derived as a surrogate to evoke hyperpnic‐induced vasodilation (MICT) and hyperventilation‐induced vasoconstriction (HIIT), which the physiological data in Table 1 indicate was achieved. Therefore, despite the prediction error associated with age‐based equations (Arena et al., 2016; Robergs & Landwehr, 2002; Shookster et al., 2020; Tanaka et al., 2001), this would not invalidate the exercise paradigms utilized. More so, although lactate and minute ventilation were not directly measured during this investigation, the P_ET_CO_2_ and CBV (i.e., PCA and MCA) values presented during the exercise protocols (Table 1) are comparable to the sea level data presented in prior work by Smith and colleagues (Smith et al., 2014). This indicates blood lactate levels would be ~1.7–2.2 mmol/min during MICT and >6.7 mmol/min during the HIIT protocols, which provides a further index the desired exercise intensity domains were achieved for each condition. Moreover, although diet was controlled across each intervention, it was not strictly controlled for prior to data collection. Participants were encouraged to consume the same quantity and type of nutrients (e.g., protein, carbohydrates, fats) at the same time prior to the start each day. Nonetheless, given the fact that there were no differences between baseline values and across the control condition, dietary concerns likely had a nominal influence on the results. Hydration status was also not strictly controlled for but was monitored throughout the entirety of the study. However, participants were given a Gatorade to consume between 8:00am to 1:30 pm and a second between 1:30 pm to 7:00 pm (Gatorade Perform, PepsiCo; 150 calories, and 35 g of sugar). As well, participants had access to water as they required. This would have kept hydration and blood plasma levels relatively consistent during each NVC assessment. Further, given that aside from where the exercise bout impacted the NVC response, all parameters were relatively similar, thus hydration likely had an inconsequential impact on the results of this investigation. Finally, all participants who volunteered to engage in this study were young healthy adults who were physically active. As a result, the findings may not be generalizable to older and/or clinically based populations.

## 5 CONCLUSIONS

In conclusion, a bout of HIIT exercise attenuated the NVC response for one hour, whereas MICT and control protocols had minimal effects. Therefore, future studies examining NVC metrics can test participants a minimum of one hour following exercise, which substantially reduces the time restrictions currently followed within the broader literature. However, it is also important to ensure other physiological variables (e.g., cardiovascular, cerebrovascular, respiratory, etc.) are controlled during data collection, as heart rate remained elevated at hour one following HIIT during the NVC assessment. Lastly, NVC measures associated with the control day were highly reliable and had minimal variation. Thus, future NVC measurements can be performed at various times between 8:00am to 7:00 pm, knowing there are nominal influences of diurnal variation within the measures.

## CONFLICT OF INTEREST

The authors declare that they have no conflicts of interest.
